# Comparative Analysis of the Lignification Process of Two Bamboo Shoots Stored at Room Temperature

**DOI:** 10.3390/plants9101399

**Published:** 2020-10-21

**Authors:** Zuying Zhang, Changtao Li, Hui Zhang, Yeqing Ying, Yuanyuan Hu, Lili Song

**Affiliations:** State Key Laboratory of Subtropical Silviculture, Zhejiang A&F University, Lin’an 311300, China; 451882@zju.edu.cn (Z.Z.); 648677@163.com (C.L.); zhangafei2020@163.com (H.Z.); yeqing@zafu.edu.cn (Y.Y.)

**Keywords:** high bamboo shoots, moso bamboo shoots, room temperature, lignification process, transcription factor, regulation

## Abstract

Two types of bamboo shoots, high bamboo (*Phyllostachys prominens*) shoots (HBSes) and moso bamboo (*Phyllostachys edulis*) shoots (MBSes), underwent a fast post-harvest lignification process under room temperature storage. To explore the mechanism of lignification in two types of bamboo shoots after post-harvest during room temperature storage, the measurement of cell wall polymers (lignin and cellulose) and enzyme activities of phenylalanine ammonialyase (PAL) and peroxidase (POD), and relative expression of related transcription networks factors (TFs) were performed. The results suggested that the lignification process in HBSes is faster than that in MBSes because of incremental increase in lignin and cellulose contents within 6 days and the shorter shelf-life. Additionally, compared with the expression pattern of lignification-related TFs and correlation analysis of lignin and cellulose contents, *MYB20*, *MYB43*, *MYB85* could function positively in the lignification process of two types of bamboo shoots. A negative regulator, *KNAT7,* could negatively regulate the lignin biosynthesis in two types of bamboo shoots. In addition, *MYB63* could function positively in HBSes, and *NST1* could function negatively in MBSes. Notably, *MYB42* may function differently in the two types of bamboo shoots, that is, a positive regulator in HBSes, but a negative regulator in MBSes. Transcription networks provide a comprehensive analysis to explore the mechanism of lignification in two types of bamboo shoots after post-harvest during room temperature storage. These results suggest that the lignification of bamboo shoots was mainly due to the increased activity of POD, higher expression levels of *MYB20*, *MYB43, MYB63,* and *MYB85* genes, and lower expression levels of *KNAT7* and *NST1* genes, and the lignification process of HBSes and MBSes had significant differences.

## 1. Introduction

Edible bamboo shoots are enlarged buds or young stems formed by the germination of the buds on the bamboo whip or stalk base. According to the different seasons of budding and unearthing, bamboo shoots can be divided into spring bamboo shoots and winter bamboo shoots. Owing to the low fat, high dietary fiber and protein with a distinctive flavor, bamboo shoots have been valued as “Forest vegetables and vegetable treasures”, which reveals their importance and popularity throughout Asian history [1]. More than 1250 bamboo species have been characterized worldwide, and most of them produce edible shoots [2]. Bamboo shoots deteriorate rapidly and can easily lose their commercial value post-harvest. Changes in the physiological environment of bamboo shoots after harvest will accelerate the lignification of the shoots [3]. Bamboo shoots undergo a rapid post-harvest process of lignifying. They deteriorate quickly, making them woody and inedible, which is the largest problem of the bamboo shoot industry at present.

The lignification process of bamboo shoots is closely related to the formation of secondary cell walls, which contain lignin and cellulose. Lignin biosynthesis and its associated regulatory mechanisms have been studied in the model plant *Arabidopsis*, woody trees, and various crops [4,5,6]. In monocots, lignin is a complex phenylpropanoid polymer that originates from the polymerization of three monolignols, p-hydroxyphenyl (H), guaiacyl (G), and syringyl (S) monomers through a peroxidase (POD) reaction, while phenylalanine ammonialyase (PAL) functions at the beginning of the monolignol biosynthetic route [7,8,9,10]. Lignin is one of the most abundant biopolymers, which serves as an important contributor to the natural stock of non-fossilized carbon on earth. More attention has been paid to the self-assembly properties of lignin and its value in its effective utilization [11,12].

In addition to its biosynthetic pathway, a complicated network of transcription factors (TFs) has been reported to be involved in lignin biosynthesis regulation; these TFs are mainly divided into two categories: NAC TFs and MYB TFs [13,14]. NAC TFs serve as “master switches” and regulate downstream levels of transcription factors, including *AtNST1* and *AtNST2*, *AtVND1* to *AtVND7*, and *AtSND1* [15,16,17,18]. MYB genes, which constitute a large family of transcription factors with multiple functions, including lignin biosynthesis, play a central role in the transcriptional regulation of secondary cell walls and have been reported to function as a link between upstream NAC TFs and downstream structural genes [19,20]. For example, *AtMYB58*, *AtMYB63,* and *AtMYB85* specifically and directly activate biosynthetic enzymes by binding to a conserved motif in their promoters [21,22]. Similar MYB transcription factors have also been characterized in other plants, such as *ZmMYB31* and *ZmMYB42* in *Zea mays* [23,24] and *PtoMYB216* in *Populus spp*. [25]. The KNAT7 gene, one of knotted *Arabidopsis thaliana* (KNAT) knotted1-like homeodomain (KNOX) gene family, acts as a transcriptional activator or repressor in *Arabidopsis* and *Populus*, which has been shown to be one of the direct targets of both SND1/VND6 [21] and MYB46 [26]. However, transcription factors related to the lignification of bamboo shoots post-harvest have not been identified.

High bamboo (*Phyllostachys prominens*) shoots (HBSes) are excellent bamboo species with tolerance to poor conditions, strong shoot growth, high yield, good quality, and strong ecological adaptability. Moso bamboo (*Phyllostachys edulis*) shoots (MBSes) have the widest distribution, the largest cultivation area, and the highest economic benefits among all bamboo plants in China. Both HBSes and MBSes are at the forefront of the cultivation area, economic benefits, yield, and ecological adaptability, and both have high research value. Considering the rapid lignification process at room temperature, exploring the occurrence of bamboo shoot lignification and its regulation mechanism has become an important issue to improve product quality and extend the shelf-life. Therefore, the post-harvest lignification process during storage in HBSes and MBSes was chosen as a topic for this study.

## 2. Materials and Methods

### 2.1. Plant Materials, Treatment, and Storage

HBSes and MBSes were harvested in a commercial orchard in Lin’an (Zhejiang, China). The shoots were transported to the laboratory on the day of harvest. Uniform shoots (basal diameter 6 cm and length 35 cm) free of visible wounding and defects were selected. Before treatment, the shoots were precooled at 8–10 °C overnight. Then, they were placed in a plastic container and stored at 25 °C for 9 days under 80–95% relative humidity and natural light. The middle part of the shoots was collected at 3-day intervals during storage at 25 °C to measure shoot firmness, as well as lignin and cellulose contents. Samples for enzyme assays and RT-qPCR analysis were rapidly frozen in liquid nitrogen and stored at −80 °C for further use. Three biological replicates were sampled for each analysis.

### 2.2. Measurement of Shoot Firmness

A texture analyzer (TA-XT2i, Stable Micro Systems Ltd., Godalming, UK) was applied to measure shoot firmness. A cylindrical probe was set with a 2 mm diameter that penetrated to a depth of 15 mm at a speed of 1 mm s^−1^. After removing the leaf sheaths, measurements were taken on opposite sides of every middle shoot. Three biological replicates were performed for firmness analysis.

### 2.3. Measurement of cellulose and lignin contents

Bamboo samples were cut into small pieces, mixed, dried in an oven at 105 °C, and ground into fine powder to pass through a 40-mesh sieve. With GB/T2677.6-94, nearly 3 g of shoot powder was continuously extracted with a benzene/ethanol (2:1, v/v) mixture for 6 h in a 92 °C water bath under a Soxhlet extractor. The residues were dried completely and used for lignin and cellulose determination. The lignin content was determined according to the method of Ju et al. (1993) [27]. About 1 g of dried powder was added to 12 M H_2_SO_4_, mixed, and hydrolyzed for 4 h at room temperature. Distilled water was added to dilute the H_2_SO_4_ to a final concentration of 1 M, and then the mixture was heated for 1 h at 100 °C. Next, the solution was cooled and vacuum filtered via a Buchner funnel. Finally, the filters were air-dried at 60 °C to constant weight for lignin measurement. The cellulose content was measured using the Kurschner–Hoffner method. Appropriately 1 g of dried powder was mixed with 25 mL of nitric acid-ethanol liquid, heated at 100 °C for 1 h, and then transferred into a Buchner funnel. The remaining solution was washed with nitric acid–ethanol liquid mixture and water successively. Finally, all mixed solutions were vacuum filtered and oven-dried at 105 ± 2 °C. The cellulose and lignin contents (%) were calculated using the following formula: (m_1_-m_2_)/m_0_ × 100%, where m_1_ is either the lignin or cellulose mass and funnel mass after oven-drying to a constant weight, m_2_ is the empty funnel mass, and m_0_ is the bamboo shoot sample mass.) Three biological replicates were performed for each treatment.

### 2.4. Enzyme Activity

All enzyme activity extraction procedures were performed at 4 °C. PAL activity was measured according to Jiang’s procedure with some modifications. Approximately 1 g of frozen sample was mixed with 5 mL of borate buffer (0.2 M, pH 8.8) containing 0.1 g polyvinylpyrrolidone (PVPP) and 5 mM β-mercaptoethanol. After centrifugation of the homogenate at 10,000× *g* and 4 °C for 15 min, 0.4 mL of the supernatant was collected and mixed with borate buffer (0.2 M, pH 8.0), consisting of 20 mM L-phenylalanine, at 30 °C for 30 min for PAL activity assay. The PAL activity was defined as one unit (U) that resulted in a change of 0.1 in OD_290_ per hour per gram.

For the measurement of POD activity, about 0.4–0.5 g of frozen sample was extracted with 5 mL of phosphate buffer (0.2 M, pH 7.8). Subsequently, the homogenized mixture was centrifuged at 8000× *g* and 4 °C for 10 min. The supernatant was collected and used for the POD activity assay. The assay mixture containing 0.5 mL of extract and 3 mL reaction mixture (100 mM, pH 6.0 PBS, 2-Methoxyphenol, and 30% H_2_O_2_) was measured at the absorbance 470 nm. One unit (U) of POD activity was defined as a change in OD_470_ per minute per gram. Three biological replicates were performed for each treatment.

### 2.5. Total RNA Extraction, Reverse Transcription, and Quantitative Real-Time (RT)-qPCR Analysis

For RT-qPCR analysis, total RNA was isolated using the E.Z.N.A^®^ total RNA kit (Omega Bio-tek, Norcross, GA, USA). The quality and concentration of RNA were detected through a NanoDrop^®^ ND-1000 (Thermo Scientific, Waltham, MA, USA). RNA integrity was further verified via electrophoresis on 1% agarose gel. After the elimination of genome DNA by gDNA eraser, about 1 µg RNA was applied for first-strand cDNA synthesis using a PrimeScript^TM^ RT Reagent Kit (Takara, Dalian, China) according to the manufacturer’s protocol. Reverse cDNA was diluted with water (1:10) for RT-qPCR analysis, which was carried out using SYBR^®^ Premix ExTaqTM II (Takara, Kusatsu, Shiga, Japan) on a CFX96 Touch Real-Time PCR System (Bio-Rad, Hercules, CA, USA). The specificity of primers was confirmed via melting curves and product sequencing before use. Data were analyzed, and the relative expression level of each gene was normalized with actin (Livak and Schmittgen, 2001) [28] and calculated using the 2^−ΔΔCt^ method. Primers for RT-qPCR analysis are listed in Table 1. All samples consisted of three biological and technical replicates.

### 2.6. Statistical Analysis

Data are presented as the mean ± SE (n = 3), and n represents the biological replicates. Excel 2013 and SPSS 20.0 statistical software were applied for statistical analysis. Student’s two-tailed *t*-tests (*, *p* < 0.05; **, *p* < 0.01; ***, *p* < 0.001) were used to evaluate significant differences between the two groups in this study. Figures were drawn with Origin 8.0 (Microcal Software Inc., Northampton, MA, USA).

### 2.7. Accession Numbers

The GenBank accession numbers for the genes identified are MYB20, PH01002092G0300; MYB42, PH01000060G0800; MYB43, PH01005828G0060; MYB63, PH01000030G0050; MYB85, PH01003093G0130; SND2, PH01001753G0040; NST1, PH01000003G1230; VND7, PH01000845G0490, KNAT7, PH01000107G0940; Actin, PH01000797G0130.

## 3. Results

### 3.1. Bamboo Shoot Surface Quality

**Figure 1:**
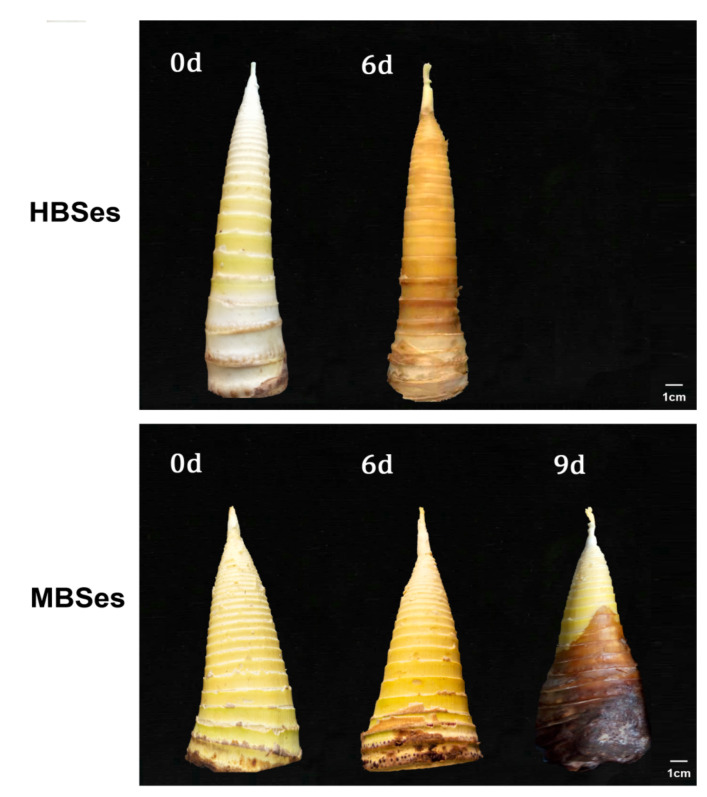
Changes in the surface quality of two varieties of bamboo shoots at room temperature. HBSes, high bamboo shoots; MBSes, moso bamboo shoots.

**Figure 2:**
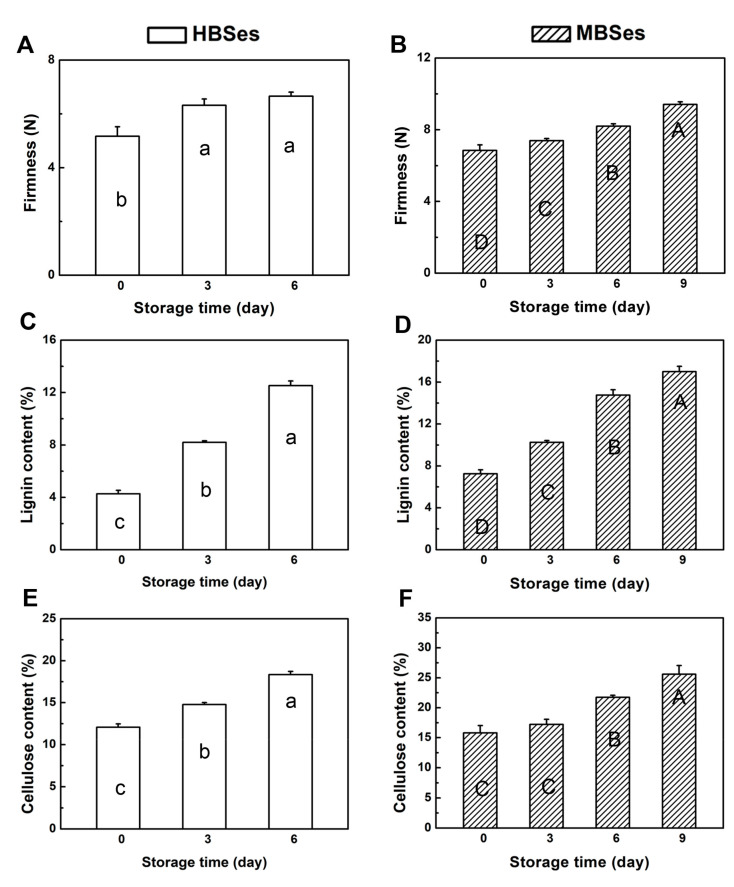
Changes in the firmness, lignin, and cellulose contents of two varieties of bamboo shoots at room temperature. (**A**) Changes in the firmness in high bamboo shoots (HBSes). (**B**) Changes in the firmness in moso bamboo shoots (MBSes). (**C**) Changes in the lignin contents in HBSes. (**D**) Changes in the lignin contents in MBSes. (**E**) Changes in the cellulose contents in HBSes. (**F**) Changes in the cellulose contents in MBSes. Error bars indicate the standard error. Different letters indicate significant differences (*p* < 0.05) among various storage times in HBSes (lowercase letters) and MBSes (uppercase letters).

The quality of HBSes declined after a 6-day storage at room temperature when shoots started to turn yellow, rot, and lose their edible qualities. However, the quality of MBSes declined after a 9-day storage (Figure 1). Degrees of firmness increased progressively in both HBSes and MBSes, among which MBSes had a higher degree of firmness (Figure 2A,B). Within 6 days, the firmness in HBSes and MBSes increased by 1.49 kg·cm^−2^ and 1.35 kg·cm^−2^, respectively (Figure 2A,B). These results show that the progression of lignification and deterioration in HBSes after harvest was faster than that in MBSes under the same storage conditions.

### 3.2. Lignin and Cellulose Contents

**Figure 3:**
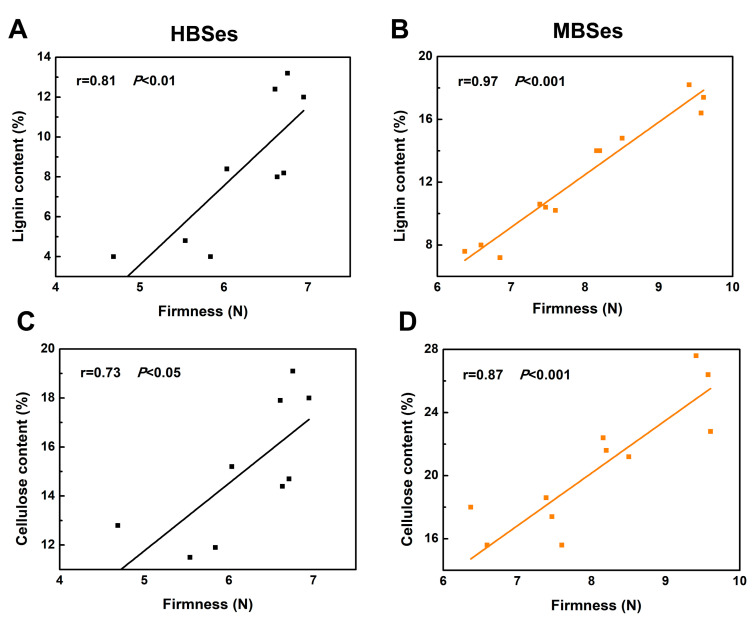
Scatterplots between firmness and lignin and cellulose contents in HBSes and MBSes at room temperature. (**A**) Linear regression analysis between firmness and the lignin content in HBSes. (**B**) Linear regression analysis between firmness and the lignin content in MBSes. (**C**) Linear regression analysis between firmness and the cellulose content in HBSes. (**D**) Linear regression analysis between firmness and the cellulose content in MBSes. Significant differences were determined using SPSS Statistics 20.0.

Lignin and cellulose are the main components of the cell wall structure in bamboo shoots. During a 6-day room temperature storage process, the increase in the lignin contents in HBSes and MBSes reached 1.94-fold and 1.03-fold, respectively (Figure 2C,D). In addition, the cellulose contents in HBSes and MBSes increased by 52% and 38%, respectively (Figure 2E,F). Additionally, a positive correlation between firmness and the lignin content (r = 0.81, *p* < 0.01; Figure 3A) and between firmness and the cellulose content (r = 73, *p* < 0.05; Figure 3B) was observed in HBSes at room temperature. Furthermore, in MBSes, there was a high positive correlation between firmness and the lignin content (r = 0.97, *p* < 0.001; Figure 3C) and between firmness and the cellulose content (r = 0.87, *p* < 0.001; Figure 3D) during storage at room temperature.

### 3.3. PAL and POD Activities

**Figure 4:**
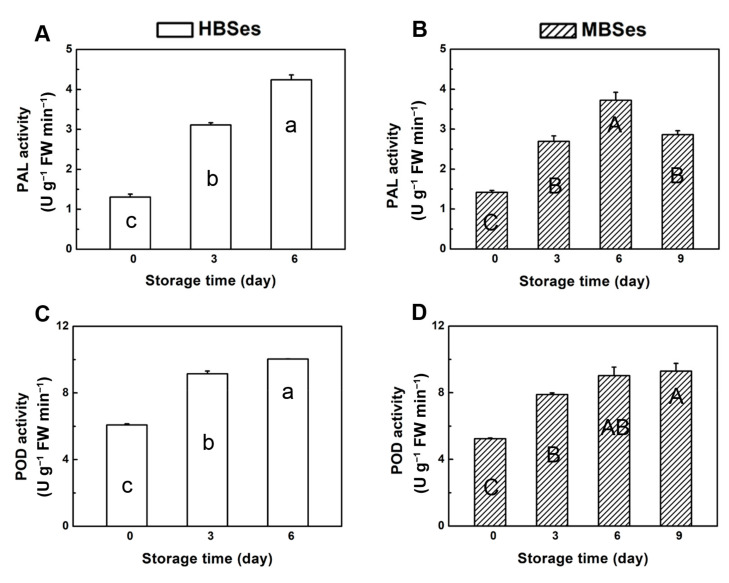
Enzyme activity of two varieties of bamboo shoots at room temperature. (**A**) Changes in the PAL activity in HBSes. (**B**) Changes in the PAL activity in MBSes. (**C**) Changes in the POD activity in HBSes. (**D**) Changes in the POD activity in MBSes. Error bars indicate the standard error. Different letters indicate significant differences (*p* < 0.05) among various storage times in HBSes (lowercase letters) and MBSes (uppercase letters).

Activities of the key lignin biosynthetic enzymes (PAL and POD) were measured during storage at room temperature. PAL and POD activities in HBSes showed an upward trend when stored at 25 °C for 6 days, and PAL increased dramatically by 225% (Figure 4A). In contrast, PAL activity in MBSes increased and reached a maximum value on day 6, followed by a gradual decrease until day 9 (Figure 4B). POD activity showed the same upward trend in MBSes, with a significant increase from 5.24 to 9.03 U kg^−1^ FW min^−1^ (Figure 4C,D).

### 3.4. Gene Expression Pattern of Several Transcription Factors

**Figure 5:**
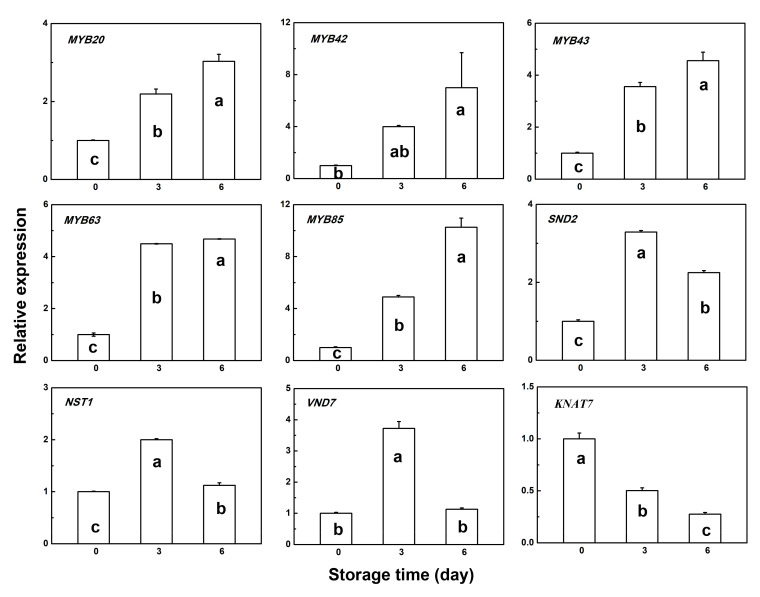
Relative gene expression patterns of transcription factors in HBSes during storage at 25 °C. Error bars indicate the standard error. Lowercase letters indicate significant difference (*p* < 0.05) among various storage times in HBSes.

**Figure 6:**
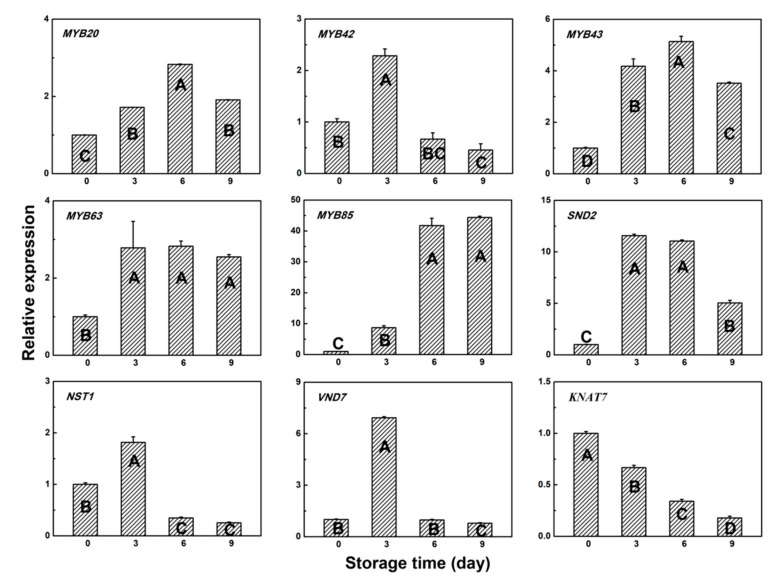
Relative gene expression patterns of transcription factors in MBSes during storage at 25 °C. Error bars indicate the standard error. Uppercase letters indicate a significant difference (*p* < 0.05) among various storage times in MBSes.

To study the lignification process in the two types of bamboo shoots during room temperature storage, expression levels of several transcription factors involved in lignin biosynthesis were measured. Expressions of *MYB20*, *MYB42*, *MYB43*, *MYB63*, *MYB85,* and *SND2* were induced in both types of bamboo shoots (Figure 5 and Figure 6). *NST1* and *VND7* expressions were significantly induced within 3 days and greatly decreased by 6 days in both types of bamboo shoots (Figure 5 and Figure 6). Furthermore, the transcript abunsdance of *KNAT7* showed a downregulated trend during storage at room temperature (Figure 5 and Figure 6). Correlation analysis between these TF expression levels and the lignin and cellulose contents in the two types of bamboo shoots showed that the expression patterns of *MYB20*, *MYB42*, *MYB43*, *MYB63,* and *MYB85* were highly consistent with both lignin and cellulose contents in HBSes, among which the *MYB85* transcript showed the same positive correlation with the lignin and cellulose contents in MBSes (Table 2). The expression levels of *MYB20* and *MYB43* in MBSes showed a positive correlation with the lignin content only (Table 2). However, the *MYB42* transcript level showed a negative correlation with the cellulose content in MBSes, which was opposite to that observed in HBSes (Table 2). Notably, the expression level of *KNAT7* was significantly negatively correlated with the lignin and cellulose contents in both HBSes and MBSes under room temperature storage; the correlation coefficients were −0.95 and −0.91 and −0.97 and −0.87, respectively (Table 2). *NST1* showed no correlation in HBSes, while a significant negative correlation between the lignin and cellulose contents was observed in MBSes (Table 2).

## 4. Discussion

### 4.1. The Effect of Room Temperature Storage on the Lignification Process of Post-Harvest Bamboo Shoots

The color changes (turning to dark yellow or browning) of the plant are the result of polyphenol oxidation [29,30]. Numerous studies have shown that PAL and POD activities increased with increasing browning [31,32]. In loquat, the significant increase in fruit firmness during ripening is a consequence of tissue lignification; its progress correlated with increases in POD activity, and the lignification biosynthesis might involve a coordinated regulation of transcription factors [33]. Most bamboo shoots emerge at the turn of spring and summer. The temperature is high, the metabolic activity of bamboo shoots is high, the aging process is fast, and results in a great loss of economic value. The two varieties of bamboo shoots underwent rapid post-harvest lignification under room temperature storage, losing their edibility and resulting in a great loss (Figure 1). The lignification process was accompanied by a deterioration in appearance, owing to the increased firmness, increased lignin and cellulose contents, and increased POD activity (Figure 2 and Figure 4). Therefore, bamboo shoots are often stored at low temperature in practical applications, which can significantly slow down the lignification process [34].

The results showed that HBSes could be stored for 6 days at room temperature, at which point, the bamboo shoots started to deteriorate, while the storage time of MBSes could be longer, i.e., 9 days (Figure 1). The difference in the lignification process of the two varieties was mainly reflected in that HBSes had a higher accumulation of lignin and cellulose than MBSes. In asparagus spears, significant increases in lignin contents were also measured in all three sections under cold storage, with the greatest increase measured in the basal section, indicating the rapid lignification process occurred in asparagus spears [35]. The reason for the difference was that the activities of POD in HBSes were higher than that in MBSes under different storage times at room temperature. The lignification process in HBSes was faster than that in MBSes under the same storage conditions, making HBSes less durable and its shelf life shorter. The result revealed that the variation of the lignification mechanism of two types of bamboo shoots after harvest was mainly due to the different degrees of firmness, different accumulation of lignin and cellulose contents, which may be due to the different enzyme activities of lignification-related POD.

### 4.2. Effects of Room Temperature Storage on the Molecular Mechanism of the Lignification Process in Two Varieties of Bamboo Shoots

The lignification process of post-harvest bamboo shoots is regulated by a complex transcriptional network comprising NAC, MYB, and other families of TFs, which have been widely studied in energy plants and model plants (e.g., *Arabidopsis*, *Eucalyptus*, *Pinus taeda*, *Populus trichocarpa*, *Antirrhinum majus*, and *Zea mays*). In this network, NAC proteins, including VND1-7 and NST1-3, were identified in *Arabidopsis* to serve as master regulators in various cell types [15,36]. EgMYB2 regulated lignin biosynthesis positively in stems when overexpressed in tobacco [4]. PtMYB1, PtMYB8, PtrMYB3, and PtrMYB20 have been reported as activators of lignin biosynthesis [37,38]. However, the regulatory mechanisms of bamboo shoot lignification are not well understood, and relevant transcription factors related to lignification have also rarely been characterized. Compared with the extensively reported TFs in energy plants and model plants, and given the important economic value of bamboo shoots, the TFs involved in bamboo shoot lignification require more detailed investigation.

In *Arabidopsis*, NAC TFs have mainly been characterized as master switches for secondary cell wall metabolism, rather than being the direct regulators of biosynthetic structural genes [39], with rare exceptions, such as AtVND7 and AtSND1 [40,41]. According to our previous report, some lignification-related TFs were chosen to explore the lignin biosynthesis of two types of bamboo shoots during storage at room temperature [42]. NST1 showed no correlation in HBSes, while a significant negative correlation between lignin and cellulose contents in MBSes was observed, revealing a different role in the two types of bamboo shoots. In *Arabidopsis*, for example, NST1 has been reported to regulate secondary wall thickening and serve as a master switch of fiber cell differentiation [22].

It is commonly recognized that MYB TFs play an important regulatory role in the lignin biosynthesis pathway. The result showed that MYB20, MYB42, MYB43, and MYB63 may be positive regulators of both lignin and cellulose biosynthesis in HBSes; MYB20 and MYB43 in MBSes had a positive correlation with the lignin content. Another MYB gene, MYB85, may be a positive regulator of both lignin and cellulose biosynthesis in both types of bamboo shoots, with high respective correlation coefficients of 0.98 and 0.98 in HBSes and of 0.95 and 0.88 in MBSes. In *Arabidopsis*, the overexpression of *AtMYB85* could increase the stem lignin content, and the overexpression of *AtMYB63* could activate lignin biosynthetic genes and induce the ectopic deposition of lignin [21,22]. Similar results have been confirmed in other plants. The same transcript level of TF *MYB42* showed a positive correlation with the cellulose content in HBSes, while a negative correlation in HBSes, and may function differently in the two types of bamboo shoots. AtMYB42 is assumed to be a repressor that negatively regulates secondary cell wall biosynthesis in *Arabidopsis* [21].

KNAT7 is considered a third-level transcriptional regulator in secondary cell wall formation, while it remains controversial as to whether it acts as a negative or positive regulator [43,44,45,46]. Our result suggests that KNAT7 may play a negative role in lignin and cellulose biosynthesis in both HBSes and MBSes under room temperature storage, with high correlation coefficients of −0.95 and −0.91 and −0.97 and −0.87, respectively.

In summary, the results suggest that some TFs may have the same positive regulatory role in the lignin biosynthesis of both types of bamboo shoots, such as MYB20, MYB43, and a negative regulatory role like KNAT7. In contrast, some TFs, such as MYB42 and NST1, may play different roles in the lignin biosynthesis of the two types of bamboo shoots, and this needs further study and verification. Consequently, our results reveal that a complicated transcriptional network constituted of various types of transcription factors might regulate lignin biosynthesis in these two varieties of bamboo shoots.

## 5. Conclusions

The results in this study showed the lignification process in the cell walls of two varieties of bamboo shoots during room temperature storage. Firmness and the lignin and cellulose contents increased, and the activities of lignification-related enzyme POD increased accordingly as the storage time progressed. Additionally, the expression patterns of lignification-related transcription factors were provided here to explain the transcriptional network involved in the lignification process in the two types of bamboo shoots during storage at room temperature further. This study provides a theoretical basis for revealing the occurrence and molecular mechanism of bamboo shoot lignification in two bamboo varieties during room temperature storage.

## Figures and Tables

**Table 1:** Primers used for reverse transcription quantitative PCR.

Gene	Forward (5′-3′)	Reverse (5′-3′)
Actin	TGAGCTTCCTGATGGGCAAG	CCTGATATCCACGTCGCACTT
MYB20	ACCCATCTCACCGTCCCAAA	TCTGCCTCCAGAGAGCTCCA
MYB42	TGGTGAAGTGGCTGCTGGAA	CCAGCAAGCTCGAGTCCCAT
MYB43	GAGTGGCCGGACACCATGTA	CATGCCTCCTGGTCAAACGC
MYB63	AGGAGGACATGCGCCTCATC	TGAAGTTGCCGCGTTTGAGG
MYB85	AGGTCGACCCGCTGGTAAAG	TAGTCGAGCAGCCAGTTCGT
SND2	AGGGTGGCCATGGTGGTAAC	CCCTCCTGTGTGCACCTCAA
NST1	GTCATCCGCGACGTCGATCT	CGGCGTTGTAGATGGCCTTG
VND7	GTACGGGCATGAGGAGCAGT	CGATCACCCTCGACCTGGAC
KNAT7	GCAGGACCTAACTGGTGCGA	TCCTGCCTGACCCTCTCCAT

**Table 2:** Correlation analysis between transcription factors and lignin and cellulose contents in high bamboo shoots (HBSes) and moso bamboo shoots (MBSes) under room temperature storage.

Variety	Value	*MYB20*	*MYB42*	*MYB43*	*MYB63*	*MYB85*	*SND2*	*NST1*	*VND7*	*KNAT7*
HBSes	Lignin	0.97 ***	0.79 *	0.93 ***	0.87 **	0.98 ***	0.52	0.09	0.02	−0.95 ***
	cellulose	0.95 ***	0.81 *	0.89 ***	0.83 **	0.98 ***	0.46	0.04	−0.03	−0.91 ***
MBSes	Lignin	0.64 *	−0.54	0.59 *	0.56	0.95 ***	0.26	−0.69 *	−0.33	−0.97 ***
	cellulose	0.52	−0.61 *	0.43	0.46	0.88 ***	0.11	−0.71 **	−0.43	−0.87 ***

Asterisks denote significant differences based on Student’s *t*-test, * *p* < 0.05, ** *p* < 0.01, *** *p* < 0.001. HBSes, high bamboo shoots; MBSes, moso bamboo shoots.
